# Malignant Peritoneal Mesothelioma: Patterns of Care and Survival in the Netherlands: A Population-Based Study

**DOI:** 10.1245/s10434-019-07803-z

**Published:** 2019-10-16

**Authors:** Nadine L. de Boer, Job P. van Kooten, Ronald A. M. Damhuis, Joachim G. J. V. Aerts, Cornelis Verhoef, Eva V. E. Madsen

**Affiliations:** 1 Department of Surgical Oncology, Erasmus MC Cancer Institute, Rotterdam, The Netherlands; 2 Department of Research, Comprehensive Cancer Organization, Utrecht, The Netherlands; 3 Department of Pulmonary Medicine, Erasmus MC Cancer Institute, Rotterdam, The Netherlands

## Abstract

**Background:**

Malignant peritoneal mesothelioma (MPM) is a rare and aggressive disease. Recently, focus has shifted toward a more aggressive and multimodal treatment approach. This study aimed to assess the patterns of care and survival for MPM patients in the Netherlands on a nationwide basis.

**Methods:**

The records of patients with a diagnosis of MPM from 1993 to 2016 were retrieved from the Dutch Cancer Registry. Data regarding diagnosis, staging, treatment, and survival were extracted. Cox regression analyses and Kaplan–Meier survival curves were used to study overall survival.

**Results:**

Between 1993 and 2016, MPM was diagnosed for 566 patients. Overall, the prognosis was very poor (24% 1-year survival). The most common morphologic subtype was the epithelioid subtype (88%), followed by the biphasic (8%) and sarcomatoid (4%) subtypes. Surgical treatment has become more common in recent years, which most likely has resulted in improved survival rates. In this study, improved survival was independently associated with hyperthermic intraperitoneal chemotherapy (hazard ratio [HR], 0.33; 95% confidence interval [CI], 0.21–0.55) and surgery with adjuvant systemic chemotherapy (HR, 0.33; 95% CI, 0.23–0.48). Nonetheless, most patients (67%) do not receive any form of anti-cancer treatment.

**Conclusion:**

This study indicated that MPM still is a rare and fatal disease. The survival rates in the Netherlands have improved slightly in the past decade, most likely due to more aggressive treatment approaches and increased use of surgery. However, most patients still do not receive cancer-directed treatment. To improve MPM management, and ultimately survival, care should be centralized in expert medical centers.

Malignant peritoneal mesothelioma (MPM) is a rare and aggressive neoplasm arising from the serosal lining of the abdominal cavity.^1^ It represents about 10–15% of all malignant mesothelioma cases, making it the second most common location.^2^ Pleural mesothelioma is far more common, representing more than 80% of cases. Other, more rare, locations are the pericardium (< 1%) and the tunica vaginalis of the testis (< 1%). The main risk factor for the development of malignant mesothelioma is asbestos exposure.^3^

Generally, MPM is predominantly known as a locally aggressive tumor. Malignant ascites and locoregional invasion cause morbidity and mortality, whereas metastatic lymph nodes (5–10%) and extraabdominal disease (3–5%) are rare. Patients experience nonspecific symptoms such as nausea, abdominal pain, weight loss, and abdominal distension.^4^ Accordingly, diagnosing MPM is difficult and often delayed. As a result, MPM is mostly diagnosed when patients are in an advanced stage of the disease, leading to poor survival rates even after extensive treatment.^5^

Due to the rarity of MPM, little is known about the epidemiology and treatment patterns on a nationwide basis. Also, no randomized studies comparing outcomes of different treatment strategies are available, implying the need for large retrospective cohort studies. Therefore, in this study, MPM incidence, patterns of care, and survival on a population-based level in the Netherlands were investigated during a 24-year period.

## Methods

### Collection of Data

Data on patients with MPM diagnosed from 1993 through 2016 were retrieved from the Netherlands Cancer Registry (NCR) after formal approval by the NCR Monitoring Committee. The NCR collects data on all patients with cancer diagnosed in the Netherlands based on notification of newly diagnosed malignancies by the national automated pathologic archive and on hospital discharge diagnoses. Information on diagnosis, staging, and treatment is extracted routinely from the medical records by specially trained NCR personnel. Information on survival status is updated annually using a computerized link with the national civil registry. For the current analysis, survival information was updated to 1 February 2019. Cause of death was not available due to privacy regulations.

Stage information was recorded according to the Surveillance Epidemiology and End Results (SEER) extent of disease (EOD) classification, distinguishing local, regional, and distant progression. Local disease is confined to the peritoneum, whereas regional disease comprises contiguous growth to adjacent organs or extension to regional lymph nodes. Distant progression may include invasion of intraabdominal organs.

For the most recent period, between 2009 and 2016, information on the site of distant metastases was available. Tumor site and histologic subtype were recorded according to the topography and morphology codes of the International Classification of Diseases for Oncology (ICD-O-3).

Since 2000, all suspected cases of malignant mesothelioma in the Netherlands are reviewed by the Dutch National Mesothelioma Panel (NMP), a group of expert pathologists and (pulmonary) oncologists.^6^,^7^ Their review process is primarily based on pathology, but when no material is available for review or no definite diagnosis can be made, the case is reviewed by three independent clinicians specialized in mesothelioma. At least two of the three specialists must independently confirm the diagnosis. Before 2000, this expert review was not performed systematically, but a similar panel of specialists has been available since 1972 to advise in diagnosing mesothelioma.^8^ Treatment information comprises coding for resection surgery, systemic chemotherapy, and local (intraperitoneal) chemotherapy.

Unfortunately, specifics regarding type of surgery, extent of cytoreduction, type of chemotherapy, and number of cycles were not available. Data about comorbidity or performance status also were not available. Asbestos exposure was not reported in the national registry.

The Netherlands comprises 92 hospitals, and cytoreductive surgery (CRS) with hyperthermic intraperitoneal chemotherapy (HIPEC) is performed in nine centers. During the entire period of this study, the country’s population grew from 15.5 million to 16.7 million inhabitants.

### Statistical Analyses

The Kaplan–Meier method was used for survival analysis, and comparisons between groups were made using log-rank test. Overall survival was calculated from the date of diagnosis until death or last follow-up visit. Patients were censored when alive at the last follow-up date. Treatment patterns were tabulated by period of diagnosis and evaluated with Chi square analyses. Multivariable Cox proportional hazards models were constructed to identify prognostic factors, and hazard ratios (HRs) with 95% confidence intervals (CIs) for these factors were calculated. Nonsignificant prognostic factors were excluded from the model using backward elimination. Two-sided *p* values lower than 0.05 were considered statistically significant. Statistical analyses were performed using STATA version 14 (StataCorp 2015. Stata Statistical Software: Release 14; StataCorp LP, College Station, TX, USA). Figures were made using R version 3.5.1 (http://www.r-project.org) and GraphPad Prism version 5.00 for Windows (GraphPad Software, San Diego, CA USA; www.graphpad.com).

## Results

### Patient Characteristics

During the study period, MPM was diagnosed for 566 patients: 420 men (74%) and 146 women (26%) (Table 1). The median age at diagnosis was 69 years (interquartile range [IQR], 62–76 years) for the men and 65 years (IQR, 54–75 years) for the women.

**Table 1:** Patient characteristics

	Subjects *n* (%)^a^	Median survival Months (IQR)	1-Year survival (%)	2-Year survival (%)	*p* value
Overall	566 (100)	4.5 (1.5–11.6)	24	15	–
Gender					
Men	420 (74)	3.6 (1.3–8.1)	17	10	< 0.001
Women	146 (26)	8.9 (2.8–33.1)	45	30	
Age (years)					
0–64	197 (35)	7.3 (2.7–26.6)	40	27	< 0.001
65–74	199 (35)	4.6 (1.7–9.5)	19	11	
75+	170 (30)	1.9 (0.9–6.9)	12	6	
EOD stage					
Local	172 (30)	5.4 (1.8–14.9)	28	17	0.102
Regional	136 (24)	5.0 (1.4–12.2)	26	15	
Distant	111 (20)	3.6 (1.1–10.8)	20	14	
Unknown	147 (26)	3.1 (1.5–9.9)	20	14	
Period					
1993–2000	166 (29)	4.5 (1.4–7.7)	17	10	0.02
2001–2008	195 (34)	3.8 (1.5–11.3)	23	13	
2009–2016	205 (36)	5.0 (1.6–18.6)	31	20	
Morphology					
Epithelioid	324 (57)	5.0 (1.2–8.0)	27	18	0.02
Sarcomatoid	14 (2)	2.0 (1.1–12.0)	29	14	
Biphasic	31 (5)	3.4 (1.8–14.9)	13	9	
NOS	197 (35)	3.6 (1.2–8.1)	20	11	
Therapy					
Chemotherapy	117 (21)	8.8 (5.0–17.1)	36	18	< 0.001
Surgery ± chemo	43 (8)	15.5 (4.7–67.1)	56	44	
HIPEC ± CRS	28 (5)	23.4 (6.9–83.6)	68	50	
Other/BSC	378 (67)	2.5 (1.1–6.7)	13	8	

*IQR* interquartile range, *EOD* extent of disease classification, *NOS* not otherwise specified, *HIPEC* hyperthermic intraperitoneal chemotherapy, *CRS* cytoreductive surgery

^a^Percentages in the subjects column do not add up to 100% due to rounding

The stage of disease was available for 74% of the cases. Local disease was reported in 30% of the cases, and in 24% of the cases, MPM had spread regionally. Distant progression was seen in 20% of the patients.

More detailed information regarding metastatic sites was available only for the latest period, between 2009 and 2016. During this period, distant progression according to the EOD classification was reported for 23.4% of the patients. Of these metastases, 42.5% were located intraabdominally, and 20% were lymph node metastases whose exact location was not specified. Therefore the true percentage of cases in which the disease had spread outside the abdominal cavity was between 8.8 and 13.5%. The extraabdominal metastatic sites were the pleura (7.6%) and the lung (2.3%).

Information about histopathologic subtype was available for 65% of the patients. Most frequently observed was the epithelioid subtype, in 89% of the cases, followed by the biphasic subtype in 8% and the sarcomatoid subtype in 4%. The diagnosis was based on histology in 89% of the cases and on cytology in 11% of the cases. In 35% of the cases, the histopathologic subtype was not specified in the pathology report and therefore not registered.

### Treatment Patterns

**Fig. 1:**
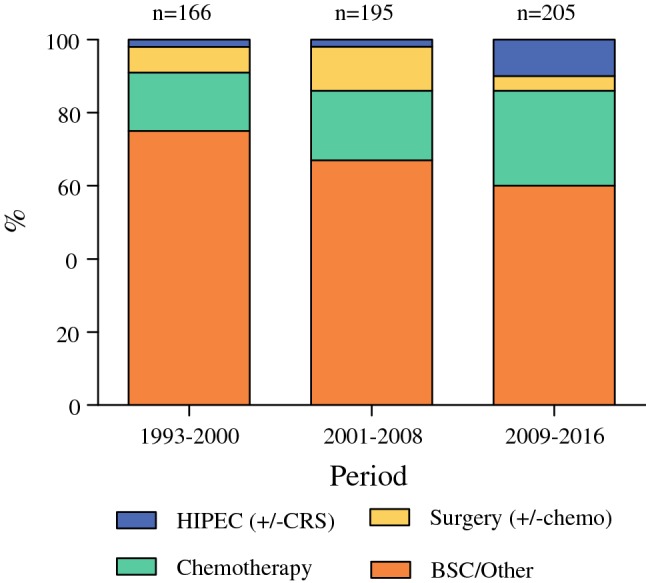
Trends in MPM treatment. *HIPEC* hyperthermic intraperitoneal chemotherapy, *CRS* cytoreductive surgery, *chemo* systemic chemotherapy, *BSC* best supportive care

**Fig. 2:**
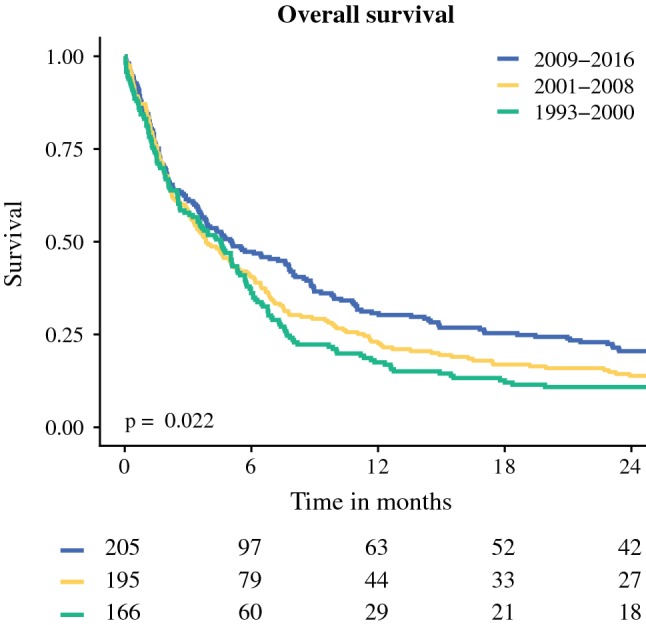
Kaplan–Meier actuarial overall survival curve

To evaluate patterns of care over time, the data were stratified into three periods: period 1 (1993–2000), period 2 (2001–2008), and period 3 (2009–2016). The treatment patterns are depicted in Fig. 1, and the corresponding survival per period is shown in Table 1 and Fig. 2. The use of systemic chemotherapy without surgery increased over time, from 16% in period 1 to 26% in period 3.

In the most recent years, treatment strategies have been more aggressive, with increased use of surgery in combination with local or systemic chemotherapy. The use of intraperitoneal chemotherapy increased, from 2% of the patients in periods 1 and 2 to 10% in period 3. Combination of surgery with systemic chemotherapy decreased in the latest years, from 12% between 2001 and 2008 to 4% between 2009 and 2016. The percentage of patients who did not receive any cancer-directed treatment declined gradually over the years, from 75 to 60%. This group however still comprises the majority of patients.

### Survival

Overall survival improved over time, with better survival between 2009 and 2016 than between 1993 and 2000 (*p* = 0.023) (Fig. 2). It was suggested that this could be an effect of treatment trends. Therefore, treatment strategies were included in the multivariable analysis. Better survival was independently associated with surgery (HR, 0.33; 95% CI, 0.23–0.48), HIPEC (HR, 0.33; 95% CI, 0.21–0.55), and systemic chemotherapy (HR, 0.61; 95% CI, 0.49–0.76) (Table 2). Female sex also was associated with better survival (HR, 0.65; 95% CI, 0.53–0.81). Age of 65–74 years at the time of diagnosis (HR, 1.55; 95% CI, 1.25–1.92) and age older than 75 years (HR, 2.00; 95% CI, 1.59–2.51) was independently associated with diminished survival outcome. Significantly worse survival was associated with the sarcomatoid (HR, 1.23; 95% CI, 0.70–2.15), biphasic (HR, 1.54; 95% CI, 1.05–2.26), and undefined morphologic (HR, 1.26; 95% CI, 1.05–1.52) subtypes.

**Table 2:** Multivariable survival analysis

	HR	95% CI	*p* value
Gender			
Men	1		
Women	0.65	0.53–0.81	< 0.001
Age (years)			
0–64	1		
65–74	1.55	1.25–1.92	< 0.001
≥ 75	2.00	1.59–2.51	< 0.001
Morphology			
Epithelial	1		
Sarcomatoid	1.23	0.70–2.15	0.476
Biphasic	1.54	1.05–2.26	0.027
NOS	1.26	1.05–1.52	0.012
Therapy			
BSC/other	1		
Chemotherapy alone	0.61	0.49–0.76	< 0.001
Surgery ± chemo	0.33	0.23–0.48	< 0.001
HIPEC ± CRS	0.33	0.21–0.55	< 0.001

*HR* hazard ratio, *CI* confidence interval, *NOS* not otherwise specified, *BSC* best supportive care, *chemo* systemic chemotherapy, *HIPEC* hyperthermic intraperitoneal chemotherapy, *CRS* cytoreductive surgery

## Discussion

This study provides insight into the epidemiology and changes in patterns of MPM treatment on a nationwide basis in the Netherlands. Analyzing data retrieved from the NCR showed that MPM is a rare disease and that prognosis still is very poor. Patients eligible to undergo surgery, HIPEC, or both have significantly better median survival rates than patients receiving systemic chemotherapy or best supportive care. Although an increasing percentage of patients receive anti-cancer therapy, the majority of patients do not. To achieve better outcome, all patients should be referred to specialized physicians for evaluation of their eligibility to undergo surgical treatment.

The patient characteristics found in this study are comparable with those described in earlier reports.^9^–^14^ Women with MPM live longer than men with MPM. It is sometimes suggested that the better survival among women is caused by a misdiagnosis of MPM because it has similarities to ovarian cancer. However, in the current cohort, most of the diagnoses were determined by expert mesothelioma panels, so this explanation seems unlikely.

What causes better survival among women remains debated. The fact that women are less often exposed to asbestos might be favorable for their prognosis because asbestos exposure has been associated with poor survival.^15^,^16^ Also some studies have reported that higher cumulative exposure to asbestos results in more aggressive mesothelioma subtypes.^17^

A recent study of 16,267 mesothelioma patients by van Gerwen et al.^18^ showed significantly more epithelial mesothelioma in female patients, thereby supporting these earlier findings. However, after correction for histology and other prognostic factors, female gender still was independently associated with better survival. This finding supports earlier theories on the protective role of circulating estrogen and estrogen receptor-beta expression in mesothelioma.^16^,^19^

Regarding tumor characteristics, morphology was shown to have prognostic value, but tumor stage was not. In earlier series, tumor morphology already was identified to be of prognostic value: the epithelioid subtype has better survival outcomes than the sarcomatoid or biphasic subtype.^14^ The finding that tumor stage does not seem to influence survival of MPM patients can be explained by the lack of an unambiguous staging system. Although efforts have been made to develop a staging system,^20^ to date, no standardized method has been implemented on a international level. Therefore, most cases of MPM are not staged according to a uniform system, which generates heterogeneous outcomes between series.^9^,^11^–^13^

This study found that 20% of patients experienced distant metastases, which is considerably more than in studies published by Liu et al. and Yan et al.^14^,^20^ Before 2009, the sites of metastases from MPM were not specifically reported in the Dutch National Cancer Registry. Analysis of the reported data during the period between 2009 and 2016 showed that metastases occurred in 23.4% of the cases. However, detailed analysis of the metastatic sites showed that the percentage of cases with spread of the disease outside the abdominal cavity was between 8.8% and 13.5%. These findings suggest that an overestimation of distant metastases has probably occurred due to the diffuse growth pattern of MPM. However, other studies reporting less than 5% distant metastases are mainly surgical series that might underestimate the number of distant metastases due to preoperative patient selection. Population-based series earlier described similar or even higher numbers of distant metastases.^11^,^21^ A uniform staging system using systematic staging procedures (radiologic and surgical), is needed for better prognostication.

The influence of various treatment strategies on survival was statistically evaluated in this analysis. Traditional treatment options for MPM are (palliative) debulking surgery and systemic chemotherapy. More recently, the focus has shifted toward CRS–HIPEC, which has shown encouraging results.^22^–^24^ However, even when CRS–HIPEC is combined with (neo)adjuvant chemotherapy, progression-free survival is known to be very poor.^25^–^28^

In the current cohort, the patients eligible to undergo surgery (with systemic chemotherapy or CRS–HIPEC) lived significantly longer than those receiving best supportive care or systemic chemotherapy alone. However, these data should be interpreted carefully because they were collected in retrospect. No specific information concerning chemotherapeutic regimens or extent of operative procedures was available. Also no general staging system was used in clinical reports.

Differences in survival between treatment strategies are partly attributable to patient selection. Unfortunately, survival analysis could not be corrected for patient selection because no performance score or information about comorbidities was available. Nonetheless, it seems likely that improved survival in recent years has been brought about by the increasing use of surgical treatment strategies.

Although the link between asbestos exposure is not as strong for peritoneal mesothelioma as for pleural mesothelioma, up to 60% of patients have been exposed to asbestos.^3^,^29^ Other environmental agents such as zeolite fibers, a mineral found in volcanic tuff, also have been associated with mesothelioma development.^30^ In addition to environmental agents, other risk factors are germline BAP1 mutations and other deleterious mutations of tumor suppressor genes.^31^,^32^ All risk factors considered, asbestos exposure seems to be the largest contributor to MPM risk. Consequently, because asbestos-related deaths are expected to keep rising in the coming years,^33^,^34^ a pressing need exists for improvement in MPM management.

An important issue in MPM is the lack of general methods for diagnosis, staging, and treatment due to the rarity of the disease. Although patients are more often treated with aggressive regimens using surgery in combination with local or systemic chemotherapy, much room still exists for improvement because the majority of patients do not receive cancer-directed treatment. Although a substantial number of patients in the best supportive care group likely were unfit to undergo treatment due to disease burden, comorbidities, or both, it also is probable that a considerable number of patients in this group were not treated properly due to a lack of knowledge or expertise.

With the implementation of CRS–HIPEC for MPM approximately a decade ago in the Netherlands, knowledge and awareness regarding this treatment option has grown under specialized physicians. This awareness, however, seems to trail behind that for other medical specialties. Surgical treatment options are not generally known, resulting in delayed referral and treatment. This also was observed by Miura et al.,^21^ who suggested that the opportunity to improve survival with surgical therapy is lost for a large number of MPM patients in the United States. This need for awareness can be partly attributed to the lack of clinical studies.

Recently, the current authors initiated a nationwide phase 2 clinical trial in the Netherlands, known as the MESOPEC trial, to assess the feasibility of adjuvant dendritic cell-based immunotherapy (DCBI) after CRS–HIPEC for patients with MPM.^35^ Earlier, DCBI showed promising results for patients with pleural mesothelioma.^36^–^38^ By simultaneously seeking attention for this clinical trial and MPM in general, awareness of treatment possibilities and the number of referrals has increased.

Because CRS–HIPEC can significantly improve survival for selected candidates, this procedure should be available for as many patients as possible. This requires expertise in patient selection and surgical treatment. Accordingly, centralization of care for MPM patients is of significant importance for achievement of further improvement in MPM care and ultimately survival. To achieve this, all MPM patients should be referred to specialized medical centers with sufficient knowledge of therapeutic options and ample experience in performing CRS–HIPEC. Preferably, these specialized centers are connected to a comprehensive research facility or university to explore and develop new therapeutic options for patients with MPM.

Sadly, the MESOPEC trial currently is the only clinical trial in the European Union exploring new interventions for patients with MPM. To make a difference and significantly improve MPM prognosis, expert medical centers should collaborate to explore new therapeutic options and standardize treatment strategies.

## Conclusion

Survival for MPM patients has improved slightly in recent years, most likely due to more aggressive (multimodal) treatment strategies. The majority of MPM patients, however, do not receive cancer-directed treatment. Considerable progress in MPM management needs to be made and can be achieved only by centralizing MPM care in expert centers.
